# Pharmacokinetic and Pharmacodynamic Evaluation of Marbofloxacin and PK/PD Modeling against *Escherichia coli* in Pigs

**DOI:** 10.3389/fphar.2017.00542

**Published:** 2017-08-21

**Authors:** Zhixin Lei, Qianying Liu, Jincheng Xiong, Bing Yang, Shuaike Yang, Qianqian Zhu, Kun Li, Shishuo Zhang, Jiyue Cao, Qigai He

**Affiliations:** ^1^Department of Veterinary Pharmacology, College of Veterinary Medicine, Huazhong Agricultural University Wuhan, China; ^2^National Reference Laboratory of Veterinary Drug Residues and MAO Key Laboratory for Detection of Veterinary Drug Residues, Huazhong Agriculture University Wuhan, China; ^3^State Key Laboratory of Agriculture Microbiology, College of Veterinary Medicine, Huazhong Agriculture University Wuhan, China

**Keywords:** *Escherichia coli*, marbofloxacin, pigs, pharmacokinetic, pharmacodynamic, PK/PD, optimal dose

## Abstract

The aim of this study was to evaluate the activity of marbofloxacin and establish the optimal dose regimens for decreasing the development of fluoroquinolone resistance in pigs against *Escherichia coli* with *ex vivo* pharmacokinetic/pharmacodynamic (PK/PD) modeling. The recommended dose (2 mg/kg body weight) of marbofloxacin was orally administered in healthy pigs. The ileum content and plasma were both collected for the determination of marbofloxacin. The main parameters of C_max_, AUC_0-24 h_, AUC, Ke, t_1/2ke_, MRT and Cl_b_ were 11.28 μg/g, 46.15, 77.81 μg⋅h/g, 0.001 h^-1^, 69.97 h, 52.45 h, 0.026 kg/h in ileum content, and 0.55 μg/ml, 8.15, 14.67 μg⋅h/ml, 0.023 h^-1^, 30.67 h, 34.83 h, 0.14 L/h in plasma, respectively In total, 218 *E. coli* strains were isolated from most cities of China. The antibacterial activity *in vitro* and *ex vivo* of marbofloxacin against *E. coli* was determined following CLSI guidance. The MIC_90_ of sensitive strains (142) was calculated as 2 μg/ml. The minimum inhibitory concentration (MIC) of HB197 was 2 and 4 μg/ml in broth and ileum fluids, respectively. *In vitro* mutant prevention concentration, growth and killing-time *in vitro* and *ex vivo* of marbofloxacin against selected HB197 were assayed for pharmacodynamic studies. According to the inhibitory sigmoid E_max_ modeling, the value of AUC_0-24 h_/MIC produced in ileum content was achieved, and bacteriostatic, bactericidal activity, and elimination were calculated as 16.26, 23.54, and 27.18 h, respectively. Based on Monte Carlo simulations to obtain 90% target attainment rate, the optimal doses to achieve bacteriostatic, bactericidal, and elimination effects were 0.85, 1.22, and 1.41 mg/kg.bw for 50% target, respectively, and 0.92, 1.33, and 1.53 mg/kg.bw for 90% target, respectively, after oral administration. The results in this study provided a more optimized alternative for clinical use and demonstrated that the dosage 2 mg/kg of marbofloxacin by oral administration could have an effect on bactericidal activity against *E. coli*.

## Introduction

*Escherichia coli* is a frequent and crucial pathogenic bacteria which can cause septicaemia, enterocolitis and diffuse peritonitis, resulting in high mortality rates, increased morbidity and devastating economic losses in the livestock industry (Burt and Reinders, 2003; Splíchal et al., 2005; Laghari et al., 2013; Ijaz et al., 2015).

Marbofloxacin (MBF) is a synthetic third-generation fluoroquinolone antibiotic developed exclusively for veterinary treatment (EMEA, 1996). With a broad spectrum of bactericidal activity, marbofloxacin acts primarily as a bactericidal antibiotic for Gram-negative pathogens, some Gram-negative pathogens and Mycoplasma (Haritova et al., 2006; Vallé et al., 2012; Tohamy and El-Gendy, 2013). It is proposed for oral or parenteral administration for the treatment of respiratory and archenteric disease in pigs or bovines, with a high bioavailability close to 100% (Ding et al., 2010). Its extensive spectrum of activity also includes infected canine pathogens such as *Staphylococcus* spp., *Proteus* spp., *Streptococcus* spp., and *E. coli*, which has been approved for treatment in pets at a dosage of 2.0 mg/kg.bw once per day via oral administration (Spreng et al., 1995; Thomas et al., 1997; Paradis et al., 2001; Yu et al., 2007).

The pharmacokinetics (PK) of MBF has been investigated in various livestock such as goats, cows, cats, sheep, pigs, and dogs (Waxman et al., 2001; Schneider et al., 2004; Albarellos et al., 2005; Ding et al., 2010; Sidhu et al., 2010), showing a high concentration in plasma and peripheral tissue, with up to 100% bioavailability, and wide and rapid distribution in tissues. Previous studies have shown MBF to have excellent pharmacokinetic characteristics such as being well-absorbed after oral and parenteral administration, higher concentrations in tissues than in the plasma, and weakly bound to plasma proteins (<10%) (Haritova et al., 2006; Andraud et al., 2011; Sun et al., 2015). MBF is distributed widely throughout the animals’ bodies, which could reach 1.6 times higher drug concentrations in the skin than in the plasma in dogs, and MBF concentrations in the plasma can remain above the MIC (>24 h) longer than the dose density (Cester et al., 1996; Schneider et al., 1996). Although some studies have reported the pharmacokinetics of MBF in animals including pigs (Ding et al., 2010; Schneider et al., 2014; Yang et al., 2016), the pharmacokinetic data of MBF in pigs plasma and intestinal content are not sufficient enough to precisely predict the efficacy of this drug.

For the evaluation of antimicrobial drugs, it is essential to optimize the dose schedule to attain clinical cures and reduce the emergence of antimicrobial drug resistance (Burgess, 1999). The pharmacokinetics-pharmacodynamics (PK/PD) integration model can reveal the relationship between antibiotics and bacterium in specific animals, and quantify the potency and efficacy of antibiotics against bacterium. Moreover, the PK/PD integration model can also prevent resistance development and provide optimal dosage strategies (Drusano, 2007; Nielsen and Friberg, 2013). As an effective tool for assessing the optimal dosage regimens, PK/PD analysis has been recommended in the development of new antimicrobial compounds by the Food and Drug Administration (FDA) and European Medicines Agency (EMA) (Yan et al., 2017).

Few researches have investigated antibiotic concentrations at the ileum content in pigs. Due to the difficulty in determining free drug concentrations at the infected site, the PK data were obtained from the plasma in previously described reports. However, it has been demonstrated that the concentration in the plasma is significantly different from that in the target sites, such as ileum content, epithelial lining fluid and interstitial fluid, which could be observed in the results of previous studies (Messenger et al., 2012; Foster et al., 2016; Wang et al., 2016; Yan et al., 2017). Thus, it is recommended to determine the drug concentrations at the target site for PK-PD modeling to achieve more rational dosage regimens.

Since there are contradictory values in PK/PD indices which have correlated with the prevention of resistant mutant selection and efficacy, great attention has been paid to specific PK/PD indices for specific pathogens and antimicrobial agents. Prior to this study, the integration of PK data in pigs with the time course activity *ex vivo* for MBF against *E. coli* had not been performed. The antibacterial activity *in vivo* of MBF against *E. coli* and PK in the ileum content were evaluated and a typical method simple-T cannulation was used to obtain the MBF concentrations in the ileum content. Moreover, the rational dosage regimen of MBF against *E. coli* was established for veterinary clinical guidance based on PK-PD integration modeling.

## Materials and Methods

### Chemicals and Reagents

Pure standard (>98% purity) MBF was purchased from Dr. Ehrenstorfer (Augsburg, Germany). The MBF bulk drug with a chemical purity of 100.3% (No. 201104005) was produced and provided by Wuhan Huishen Biotechnology, Co., Ltd. All chemical agents for this analysis were of the high performance liquid chromatography (HPLC) grade and other organic solvents were of analytical grade. For testing the susceptibility of these bacteria to MBF, each isolate was sub-cultured at least three times in LB broth (Luria-Bertani) and LB agar (Luria-Bertani agar; Qingdao Hai Bo Biological Technology, Co., Ltd, Shangdong, China).

### Animals

Eight healthy pigs including four males and four females, weighing 15–20 kg and aged 4–5 weeks, were used for this study. These animals were placed in separate pens with free access to water, and no antibiotic feed premixes. Pigs were fed for 7 days to acclimatize prior to the study. The study was approved by the Ethical Committee of the Faculty of Veterinary Medicine at Huazhong Agricultural University. All animal care and experimental protocols were conducted in accordance with the Guide for the Care and Use of Laboratory Animals of Hubei Provincial Laboratory Animal Public Service Centre (permit number SYXK 2013-0044).

### Bacterial Strain Isolation

Two hundred and eighteen *E. coli* strains were isolated from pigs in most provinces of China (Anhui, Henan, Hubei, Jiangxi, and Guangzhou) between 2015 and 2017. According to the MIC_90_ values of strains, the *E. coli* HB197 strain, with a MIC similar to MIC_90_, was selected to study the antimicrobial activity of MBF *in vitro*. *E. coli* ATCC 25922 strain was used as a reference strain for antibiotic susceptibility determination. The species of isolates was identified by polymerase chain reaction (PCR). Prior to testing the MIC, each isolate was sub-cultured at least three times in LB and LBA.

### Antimicrobial Susceptibility Testing

Susceptibility determination of MBF against *E. coli* was performed using the agar dilution method in accordance with the CLSI recommendations from a previously described report. Strains (2–4 μl, about 10^8^ CFU/ml) were inoculated onto LBA agar plates containing newborn calf serum, with twofold serial dilutions of marbofloxacin (0.0625–32 μg/ml). The strains over 32 μg/ml were screened to expand the range of twofold dilutions of MBF. Plates of strains were incubated for 48 h at 37°C. MICs were determined as the lowest drug concentrations that caused complete growth inhibition (100%). *E. coli* (ATCC 25922) was used as the quality control (QC) strain to verify the results of the susceptibility testing.

### Determination of MIC, MBC, and MPC

The MIC of 218 *E. coli* was determined with the agar dilution method in accordance with the CLSI recommendations (Jorgensen, 1993; Lei et al., 2017a). Strains of *E. coli* (2–4 μl, about 10^8^ CFU/ml) were inoculated onto LBA agar plates with twofold serial dilutions of MBF (0.0625–32 μg/ml). Plates of strains were incubated for 48 h at 37°C. MIC was the lowest concentration of MBF where visible bacterial growth was inhibited.

A 100 μl suspension from 96-well plates of MBF where the MIC value was determined by the broth dilution method, according to the guidance of clinical and laboratory standards institute (CLSI), was diluted 10-fold or more with LB and then 10 μl of each suspension dilution was spread and counted on the LBA plates for 48 h at 37°C. MBC was the lowest concentration of MBF inhibiting 99.9% bacterial density of *E. coli*.

Then, 10^10^ CFU/ml of concentrated *E. coli* (HB197) was prepared to determine the MPC on LBA plates (Blondeau et al., 2010). In addition, the suspension was spread onto LBA, including serial dilutions of MBF (1–32 MIC); the MPC was defined as the lowest concentration inhibiting bacterial growth for 96 h at 37°C.

### Bacterial Growth Curves after Different Concentrations of MBF *In Vitro* and *Ex Vivo*

The HB197 isolate was chosen to determine the growth curve under optical density (OD_600__nm_). The OD_600__nm_ values were determined from the LB and ileum content including HB197 at different points: 0, 2, 4, 6, 8, 10, 12, and 24 h.

According to the MIC of MBF against HB197 in MIC value (2 μg/ml), LBA plates were prepared with different MBF concentrations ranging from 1/4 to 32 MIC. From the bacteria-containing fluid, 100 μl was diluted with normal sterile saline (10^-1^ to 10^-5^ dilution ratio), then aliquots of the last four diluted samples were dropped onto the LBA plates at 0, 2, 4, 6, 8, 10, 12, and 24 h of culture, after which, samples were incubated for 48 h at 37°C.

For the *ex vivo* time-killing curves (bacterial growth curves after different concentrations of MBF), the bacteria (10^6^ CFU/ml) were co-incubated with ileum content samples obtained from pigs at different points (0, 0.25, 0.5, 1, 2, 4, 6, 8, 10, 12, and 24 h) after treatment with 2 mg/kg MBF by oral administration. The *ex vivo* time-killing curve *in vitro* was fitted to a PD model with the hypothesis of a decrease in MBF concentration based on incubation time with the inhibitory sigmoid E_max_ model.

### Dose Administration and Experimental Design

#### Pharmacokinetic Experimental Design in Plasma

Eight healthy (bisexual and half) pigs weighing 15–20 kg and aged 4–5 weeks were used for this study. All pigs received MBF by oral administration at a dose of 2 mg/kg. Blood samples (5 ml) were collected at 0.25, 0.5, 1, 2, 4, 6, 8, 10, 12, 24, 36, 48, and 72 h after oral administration.

#### Insertion of Ileac Cannulation and Sample Procedures

Cannulation was exteriorised on the right side of the pig between the last two ribs (Wubben et al., 2001). Anaesthesia was administered intravenously with 15 mg/kg ketamine. Cannulation was composed of 10 cm medical grade rubber plastic tubing (acetal homopolymer resin) with 2–3 cm inner–outer diameters. The large diameter end of the tubing was hand-tooled to provide a flanged end with a concave inner surface to conform to the shape of ileum. All unthreaded areas on the cannula were hand-finished to smooth the surfaces and edges where the cannula would make contact with the tissues. Pigs were recovered well for 2 weeks and kept in warm and comfortable rooms until the official test. Ileum contents (10–20 g) were collected into tubes from the ileac cannulation at 0.25, 0.5, 1, 2, 4, 6, 8, 10, 12, 24, 36, 48, and 72 h after oral administration of a dose of 2 mg/kg.

#### Blood and Ileum Contents Treatment

Blood samples collected with an anticoagulant were centrifuged for 10 min at 3000 rpm to obtain the plasma. Then, 0.5 ml plasma samples were selected and 2 ml of dichloromethane was added to the tubes, being vortexed for 2 min and centrifuged at 5000 rpm for 10 min. The above procedure was repeated twice. The dichlormethane phase was transferred to a clean tube and evaporated under nitrogen in thermostat water bath with 60°C. A sample of 0.5 ml of the mobile phase was added to the dried tube to dissolve the sample. The obtained samples were filtered through membrane filters with 0.22 μm pore size and analyzed by HPLC.

The vortexed ileum content (0.5 g) was extracted in sealed 50 ml tubes by shaking for 10 min and added 10 ml dichloromethane, being vortexed for 2 min and centrifuged at 5000 rpm for 10 min. The above procedure was repeated twice. The dichlormethane phase was transferred to a clean tube and evaporated under nitrogen in thermostat water bath with 60°C. 0.5 ml of mobile phase was used to dissolve the sample in the dried tube. The obtained samples were filtered through membrane filters with 0.22 μm pore size and analyzed by HPLC.

#### HPLC Conditions for MBF and Pharmacokinetic Analysis

A C18 reverse-phase column (250 mm × 4.6 mm, i.d., 5 μm, Agilent, United States) was used for HPLC, which was performed with a 295 nm detection wavelength at 30°C. The mobile phase consisted of acetonitrile (phase A) and 0.1% formic acid (phase B) (v:v, 82:28) with 1 ml/min flow rate of mobile phase. The HPLC method validations of MBF in plasma and ileum content were analyzed with external standard method. The MBF concentrations in the range of 0.05–20 μg/ml in plasma and ileum content, and the identical concentrations MBF standard were detected by HPLC to obtain recovery, linear regression curve and coefficient of variation.

Pharmacokinetic parameters were calculated from plasma and ileum content MBF concentrations by WinNonlin software (version 5.2.1, Pharsight Corporation, Mountain View, CA, United States). Drug concentrations were plotted on semi-logarithmic graphs to choose the appropriate PK models. The PK parameters, including C_max_, T_max_, AUC, and so on were obtained by least squares regression analysis and calculated by WinNonlin software.

#### PK/PD Integration Analysis

For antibiotics whose pharmacokinetic or pharmacodynamic parameters represented concentration dependent, the PK/PD index should be AUC_0-24 h_/MIC and C_max_/MIC (Andraud et al., 2011; Yohannes et al., 2015). The AUC_0-24 h_/MIC and C_max_/MIC were selected as the combined PK/PD parameters which were calculated in each dose of the time-killing curve. The inhibitory sigmoid E_max_ model was used to analyze the integration of AUC_0-24 h_/MIC ratio *in vitro* and bacteria count change (CFU/ml) in ileum contents during 24 h incubation with WinNonlin software (Aliabadi and Lees, 2001, 2002; Aliabadi et al., 2003). The model equation was described as shown in Eq. 1:

$${\text{E=E}}_{\max}-\frac{({\text{E}}_{\text{max}}-{\text{E}}_{0})\cdot {\text{C}}^{\text{N}}}{{\text{C}}^{\text{N}}{\text{+EC}}_{\text{50}}^{\text{N}}}$$

E denotes the effect of antimicrobial agent measured as the log_10_ difference in bacterial number before and after 24 h incubation *in vitro*, E_0_ and E_max_ indicate the changes in log10 difference between 0 and 24 h in the control samples and those containing MBF samples, EC_50_ shows the AUC_0-24 h_/MIC value reached at 50% of the E_max_, C denotes the AUC_0-24 h_/MIC ratio, and N indicates the Hill coefficient.

#### Doses Estimation

The following formula was used to estimate dosages in different magnitudes of efficiency (E = 0, no change in bacterial count, E = -1, 99.9% reduction in count, E = -3, 99.99% reduction) to deduce an optimal regimen.

$$\text{Dose}=\frac{(\text{AUC/MIC})\cdot {\text{MIC}}_{\text{90}}\cdot \text{CL}}{fu\cdot \text{F}}$$

AUC/MIC indicates the targeted end-point for optimal efficacy, MIC denotes the minimum inhibitory concentration, CL shows clearance per day, *fu* indicates the free fraction of the drug in plasma, ignoring minimal binding, and F denotes the bioavailability.

The daily dose was calculated by Monte Carlo Simulations in Oracle Ball (Oracle Corporation, Redwood Shores, CA, United States).

### Statistical Analysis

MIC_90_ was calculated by using SPSS software, and statistical analysis was performed with Student’s *t*-test and Bonferroni revision to compare the parameters of each group. *P* < 0.05 was considered to indicate statistical significance of the difference.

## Results

### MIC Distribution of *E. coli*

**FIGURE 1:**
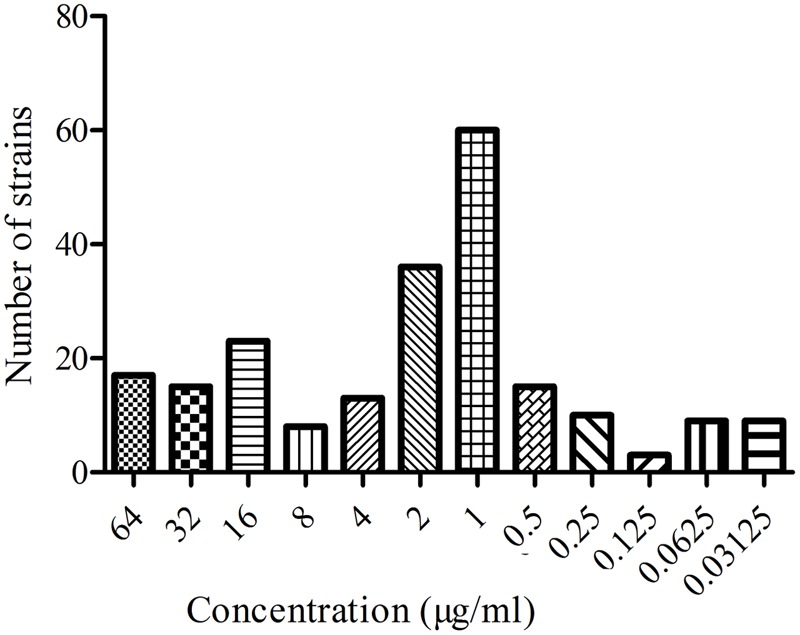
The MIC distribution of MBF against 218 *E. coli*.

The MIC distribution of the 218 *E. coli* strains to MBF are shown in the **Figure 1**. Based on the CLSI breakpoints guidance document M 100-S23, 142 susceptible *E. coli* isolates (MIC ≤ 2 μg/ml) were selected. The MIC_50_ and MIC_90_ of these susceptible strains were 0.5 and 2 μg/ml, respectively. According to the MIC_90_ values of sensitive strains, an *E. coli* (HB197) whose MIC was similar to MIC_90_ was selected and used to study the antimicrobial activity of MBF both *in vitro* and *ex vivo*.

### MIC, MBC, and MPC of MBF against *E. coli* HB197

The MIC and MBC of MBF against HB197 were 2 and 4 μg/ml in LB, and 2 and 4 μg/ml in pig ileum content, respectively. The ratios of MBC/MIC were both 2. In addition, the MPC of MBF against HB197 was 8.19 μg/ml.

### *In Vitro* and *Ex Vivo* Antimicrobial Activity of MBF

**FIGURE 2:**
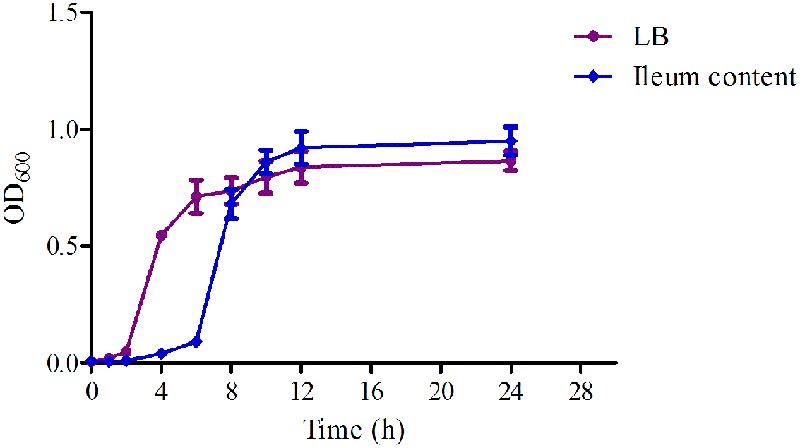
The growth-time curves HB197 *in vitro* and *ex vivo*.

The time-growth curves of HB197 *in vitro* and *ex vivo* are shown in **Figure 2**. The logarithmic phases of HB197 in LB and ileum content were 2–12 h and 4–12 h, respectively. The time to reach logarithmic phase of HB197 in LB was quicker than that in ileum content. However, the total bacterial amount of HB197 in ileum content was higher than that in LB.

**FIGURE 3:**
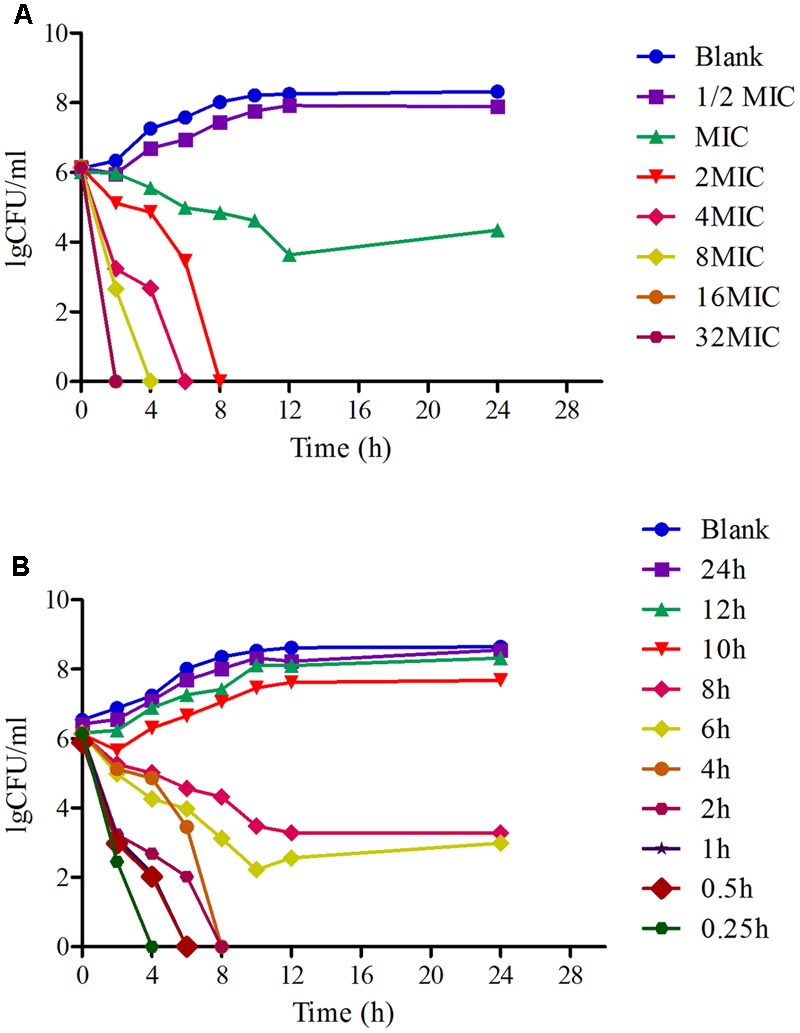
The time-killing curves of MBF against *E. coli in vitro* (LB) and *ex vivo* (ileum content). **(A)** Presented the curve *in vitro*, **(B)** presented the curve in *ex vivo*.

The time-killing curves of MBF against HB197 *in vitro* and *ex vivo* are shown in **Figures 3A,B**. Obviously, MBF displayed a concentration-dependent bactericidal activity based on the characteristic of curves *in vitro* and *ex vivo*. In the curves, there was a positive correlation between the bactericidal effect of MBF and HB197 both *in vitro* and *ex vivo*. At the same time, according to the profiles shown in **Figures 3A,B**, after exposure to the 1 MIC of MBF for 12 h, the bacterial CFU was significantly decreased, but could still recover growth. Moreover, after exposure to MBF concentrations higher than 1 MIC for 8 h, bacterial CFU were significantly decreased to undetectable levels (<30 CFU). The time-killing characters *in vitro* and *ex vivo* were similar to each other. These results also revealed that MBF was a typically concentration-dependent action both *in vitro* and *ex vivo* and that a 2 MIC concentration of MBF could completely eliminate *E. coli* after 8 h.

### Pharmacokinetic Analysis of MBF in Plasma and Ileum Content

**FIGURE 4:**
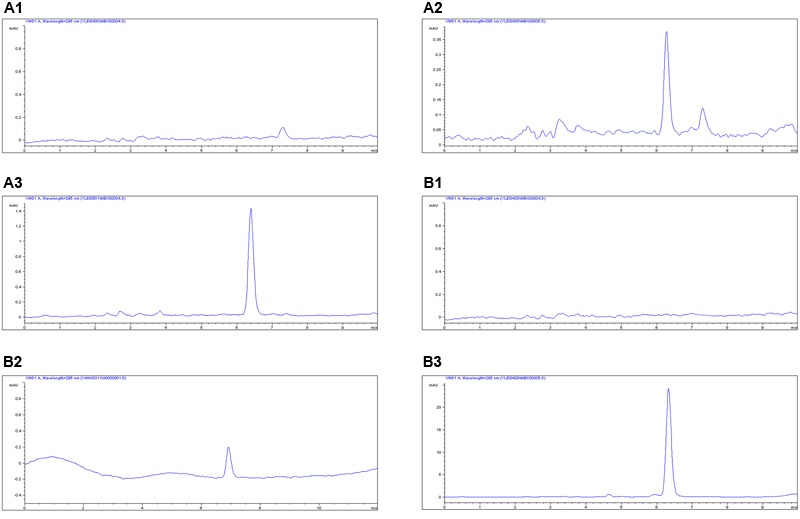
The HPLC method for MBF quantification in plasma and ileum content. The representative HPLC chromatograms of plasma were shown: **(A_1_)** blank sample, **(A_2_)** plasma sample at the LLOQ of 0.05 μg/ml, **(A_3_)** plasma sample after oral administration of MBF at the point of 1 h; the representative HPLC chromatograms of ileum content were shown: **(B_1_)** blank sample, **(B_2_)** ileum content sample at the LLOQ of 0.05 μg/ml, **(B_3_)** ileum sample after oral administration of MBF at the point of 1 h, the MBF at the retention time of 6.5 min.

The proposed methods of HPLC were suitable for MBF quantification in plasma and ileum content. These showed specificity and recovery ratios of over 92% in the plasma and 84% in the ileum content, in accordance with the veterinary drug residue guidelines of the Agriculture department and United States Pharmacopeia (Gad, 2014; Lei et al., 2017b), with a good linear relationship from 0.05 to 20 μg/ml. The chromatograms in **Figures 4A,B** showed the blank **Figure 4A**_**1**_, LLOQ **Figure 4A**_**2**_ and measured samples **Figure 4A**_**3**_ in plasma, and blank **Figure 4B**_**1**_, LLOQ **Figure 4B**_**2**_, and measured samples **Figure 4B**_**3**_ in ileum content, respectively. These indicated that the proposed methods for MBF detection in plasma and ileum content were specific and accurate. The lower limit of determination (LLOD) in plasma and ileum content were both 0.025 μg/ml, the LLOQ were both 0.05 μg/ml, and the MBF was detected at the retention time of 6.5 min.

**FIGURE 5:**
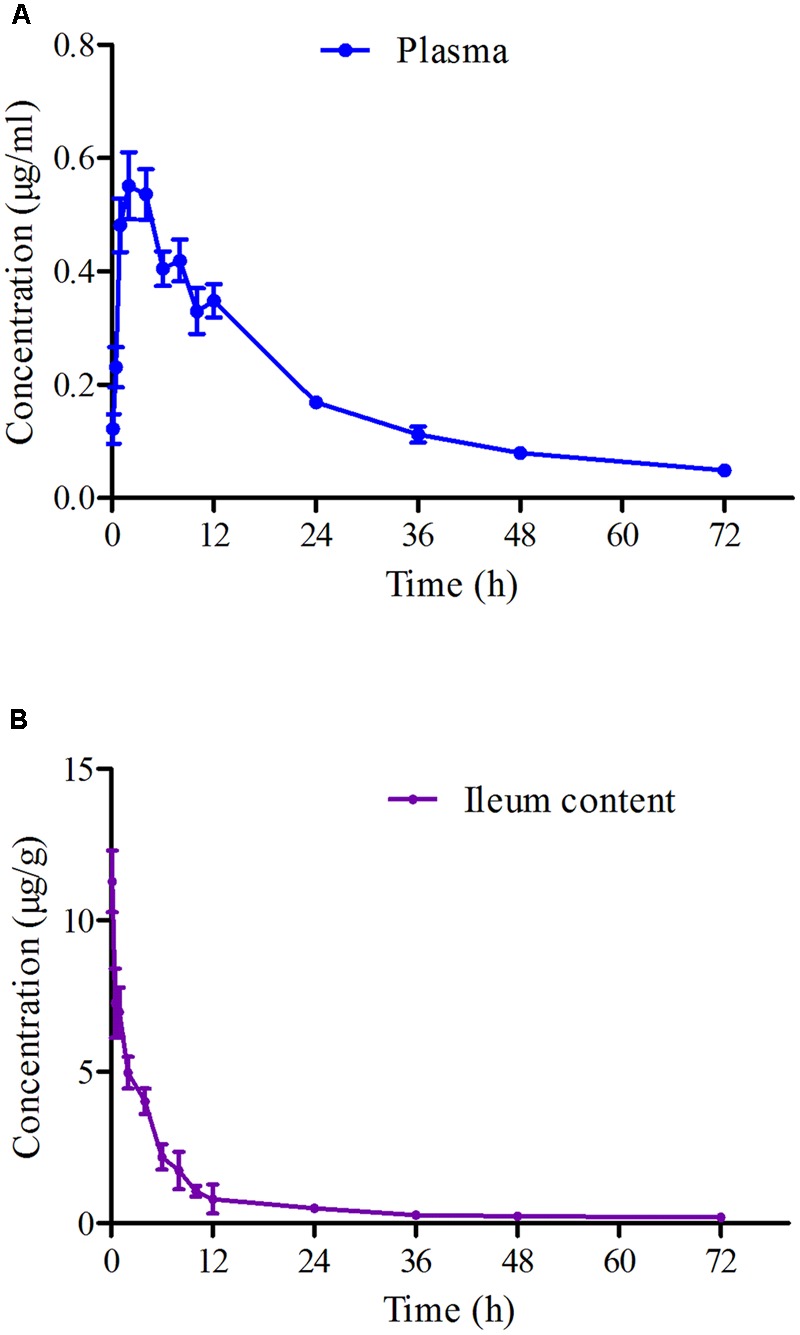
The curves of MBF concentration-time in plasma and ileum content of pigs at a dose of 2 mg/kg after oral administration at 0.25, 0.5, 1, 2, 4, 6, 8, 10, 12, 24, 36, 48, and 72 h. **(A)** Represented the curve in plasma; **(B)** represented the curve in ileum content.

The mean ± SD of MBF concentration-time profiles are shown in **Figures 5A,B** after oral gavage administration, and the main PK parameters are shown in **Table 1** using non-compartment model both in plasma ileum content. The results in **Table 1** showed that the main parameters of C_max_, AUC_0-24 h_, AUC, Ke, t_1/2ke_, MRT and Cl_b_ 11.28 μg/g, 46.15, 77.81 μg h/g, 0.001 h^-1^, 69.97 h, 52.45 h, 0.026 kg/h in ileum content, and 0.55 μg/ml, 8.15, 14.67 μg h/ml, 0.023 h^-1^, 30.67 h, 34.83 h, 0.14 L/h in plasma, respectively (**Table 1**).

**Table 1:** The main pharmacokinetic parameters mean ± SD in pigs after a single oral dose of MBF (2 mg/kg.bw).

Parameters	Units	Plasma	Ileum content
Ke	h^-1^	0.023 ± 0.006	0.001 ± 0.0003
t_1/2ke_	h	30.67 ± 4.75	69.97 ± 6.43
Cl_b_	L/h or kg/h	0.14 ± 0.03	0.026 ± 0.002
AUC	μg⋅h/ml or μg⋅h/g	14.67 ± 2.36	77.81 ± 6.38
AUC_0-24 h_	μg⋅h/ml or μg⋅h/g	8.15 ± 0.94	46.15 ± 3.62
C_max_	μg/ml or μg/g	0.55 ± 0.17	11.28 ± 1.43
MRT	h	34.83 ± 4.33	52.45 ± 5.46

*Ke, presented elimination rate constant, t_*1/2ke*_, presented elimination half-life, Cl_*b*_, presented body clearance, AUC, presented area under the curve, AUC_*0*-*24 h*_, presented area under the curve from 0 to 24 h, C_*max*_, peak concentration, MRT, presented mean residence time.*

### PK-PD Integration Modeling

**FIGURE 6:**
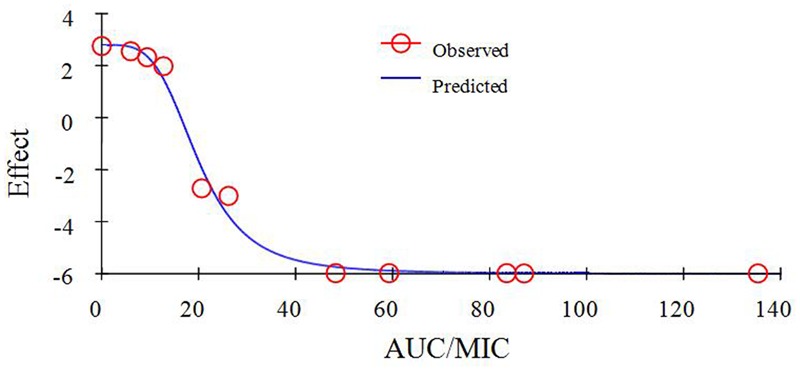
Plots of *ex vivo* AUC/MIC ratios versus the amount difference of *E. coli* HB197 within 24 h.

As a concentration-dependent action, the selected PK/PD parameters achieved from PK data *in vivo* combined with MIC and MPC *ex vivo* are shown in **Table 2**. The ratios of C_max_/MIC, C_max_/MPC, AUC_0-24 h_/MIC and AUC_0-24 h_/MPC were 5.64, 1.37, 23.08, and 5.63, respectively, based on PK/PD data in ileum content (**Table 1**). *Ex vivo* antibacterial activity of MBF against *E. coli* (HB197) was determined in ileum content samples collected before and at 0.25, 0.5, 1, 2, 4, 6, 8, 10, 12, 24 h after oral administration. The relationship between antimicrobial efficacy and the *ex vivo* PK/PD parameter of AUC_0-24 h_/MIC ratios were simulated by using the inhibitory sigmoid E_max_ model. The model parameters of the Hill coefficient N, E_0_, E_max_, and AUC_0-24 h_/MIC values are shown for three levels of growth inhibition in **Table 3** and **Figure 6**. The values of the AUC_0-24 h_/MIC ratio needed for bacteriostatic activity (E = 0), bactericidal activity (E = -3), and bacterial elimination (E = -4) were 16.26, 23.54, and 27.18 h, as shown in **Table 3**.

**Table 2:** The main PK/PD integration parameters for MBF in ileum content after a single oral dose of MBF (2 mg/kg).

Parameters	Units	Mean ± SD
C_max_/MIC	–	5.64 ± 0.64
C_max_/MPC	–	1.37 ± 0.04
AUC_0-24 h_/MIC	h	23.08 ± 3.25
AUC_0-24 h_/MPC	h	5.63 ± 0.73

**Table 3:** The main parameters of PK/PD modeling of MBF *ex vivo* after a oral dose 2 mg/kg in pigs.

Parameters	Units	Mean ± SD
E_max_	LgCFU/ml	2.56 ± 0.27
E_0_	LgCFU/ml	-6.06 ± 0.34
E_max_-E_0_	LgCFU/ml	8.59 ± 0.98
EC_50_	h	19.93 ± 2.12
N	–	3.88 ± 0.76
AUC_0-24 h_/MIC for bacteriostatic (E = 0)	h	16.26 ± 2.32
AUC_0-24 h_/MIC for bactericidal (E = -3)	h	23.54 ± 3.15
AUC_0-24 h_/MIC for eradication (E = -4)	h	27.18 ± 3.45

*E_*max*_, presented the Lg change in bacterial counts of blank sample, E_*0*_, presented the maximal antibacterial effect, EC_*50*_, presented the value to achieve 50% maximal antibacterial effect, N, presented the Hill coefficient, Hill, the dose-response curve slop.*

### Estimation of Doses

**FIGURE 7:**
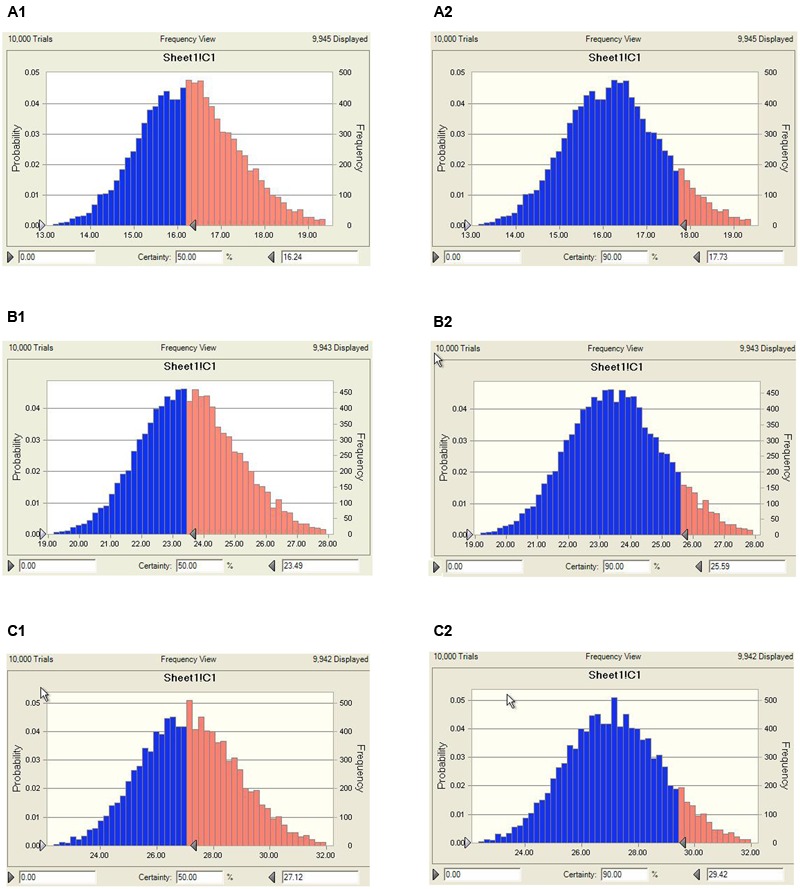
Distribution of predicted population doses (AUC_0-24 h_/MIC) of MBF against *E. coli* (HB197). **(A_1_)** Presented the predicted population dose for bacteriostatic at 50% target, **(A_2_)** presented the predicted population dose for bacteriostatic at 90% target, **(B_1_)** presented the predicted population dose for bactericidal at 50% target, **(B_2_)** presented the predicted population dose for bactericidal at 90%, **(C_1_)** presented the predicted population dose for elimination at 50% target, **(C_2_)** presented the predicted population dose for elimination at 90%.

The predicted once daily doses were shown in **Table 4** according to the AUC_0-24 h_/MIC ratios and Cl_b_ for these three levels of antibacterial activity calculated from the PK/PD integrating model and the distribution of *ex vivo* MIC using Monte Carlo Simulations in Oracle Crystal Ball. The distribution of predicted population dose (AUC_0-24 h_/MIC) values of MBF curing *E. coli* for 50 and 90% targets could be observed, respectively, in **Figure 7**. In this study, based on the dose equations, the predicted doses for bacteriostatic, bactericidal and elimination activity of MBF against *E. coli* over 24 h were 0.85, 1.22, and 1.41 mg/kg.bw for 50% target, respectively, and 0.92, 1.33, and 1.53 mg/kg.bw for 90% target, respectively in **Table 4**.

**Table 4:** The predicted daily doses of MBF curing *E. coli.*

Predicted doses (mg/kg.bw)	Target ratios	
	50%	90%
Bacteriostatic (E = 0)	0.85	0.92
Bactericidal (E = -3)	1.22	1.33
Eradication (E = -4)	1.41	1.53

## Discussion

As a new fluoroquinolone, MBF had complete absorption, and high drug concentrations in ileum content, which was the target site infected by *E. coli* after oral administration, and high antimicrobial activity *in vitro* and *ex vivo*. However, due to the overuse and misuse of antimicrobial agents, the resistance of the pathogenic bacterium to fluoroquinolone or other drugs was increasingly more serious (Burgess, 1999). An increasing number of studies about *E. coli* resistance to fluoroquinolone including MBF have been reported in most countries, such as Europe, the United States, China, and so on (Liu et al., 2013, 2015; Cheng et al., 2014; El Garch et al., 2017). The cross-resistance to similar antibiotics was widespread, and the resistance mechanisms were complex. Thus, it was crucial to reasonably use and effectively manage these new generational antibacterial agents, and necessary to establish an optimal dosage for MBF curing *E. coli* in this study.

In this study, 218 isolated *E. coli* strains were selected for PD research, such MIC, MBC, MPC and time-killing curve evaluations when exposed to MBF. In total, 142 of all selected strains were screened as sensitive populations according to the CLSI breakpoint guidance document M100-S23 (MIC ≤ 2 μg/ml) and other published reports (Andraud et al., 2011). The MIC values were in the range from 0.03215 to 64 μg/ml in whole strain populations, and 0.03125 to 2 μg/ml in the sensitive populations, respectively. The MBF-resistant *E. coli*, which had been found in many other reports, were also identified (Pellet et al., 2006; Andraud et al., 2011); the results in this study were similar to those in published reports. The mechanism of resistance of *E. coli* to MBF may be the abuse of antibiotics and cross-resistance reduced by other earlier fluoroquinolone drugs (Huguet et al., 2013; Sato et al., 2013). In the sensitive *E. coli* populations, the MIC_50_ and MIC_90_ were 0.5 and 2 μg/ml, respectively (**Figure 2**). These results revealed that MBF had high antibacterial activity *in vitro*. A highly pathogenic clinical isolate, HB197, was selected in accordance with the pathogenicity experiment in mice (data not shown) and MIC_90_. The MIC values and growth curves of HB197 in LB and ileum content showed no significant difference (**Figure 1**), and the time-killing curves *in vitro* showed that MBF was bactericidal against *E. coli*, with a concentration-dependent type (**Figure 3**). The 2 MIC concentration of MBF could completely eliminate *E. coli* after 8 h, with results similar to those previously described by Pellet et al. (2006) and Andraud et al. (2011), and other fluoroquinolone-like Enrofloxacins against *E. coli* in the reports (Wang et al., 2016). According to the profiles of **Figure 3B**, the drug concentration of MBF at the different time points from 0.25 to 4 h achieved from the plasma in PK study, had strong bactericidal effect within 8 h. Moreover, it also had bacteriostasis effect at the time points from 6 to 8 h. All of these results demonstrated that MBF had strong antibacterial activity against *E. coli in vitro* and in the ileum.

As far as we know, PK/PD integration modeling was an effect approach to dose titration studies for selecting rational dosage regimens in veterinary medicine (Toutain and Lees, 2004). Furthermore, the measurement at the infection site for PK and PD was a preferred method to analyze and correlate PK/PD modeling (Liu et al., 2002; Mouton et al., 2008). The PK of drug concentrations was investigated in serum as the *ex vivo* data in most published PK/PD modeling studies, and there were also studies for MBF against *Haemophilus parasuis* and *Pasteurella multocida* in beagle, sheep, calves, turtles, broiler chicken, pigs, and so on (Sidhu et al., 2010; Vallé et al., 2012; Potter et al., 2013; Qu et al., 2015; Sun et al., 2015; Yohannes et al., 2015). However, the PK of MBF in the ileum content which was the target infection site by *E. coli* of pigs was the first to investigate using ileac cannulation. The ileac cannulation *in vivo* could keep target animals in a normal physiological state; this state for animals had the advantage of obtaining a more accurate concentration for PK data (Garrison et al., 2002).

The PK data in plasma after oral administration of the recommended dose of 2 mg/kg for C_max_, AUC_0-24 h_, AUC, Ke, t_1/2ke_, MRT and Cl_b_ were 0.55 μg/ml, 8.15, 14.67 μg⋅h/ml, 0.023 h^-1^, 30.67 h, 34.83 h, 0.14 L/h, respectively (**Table 1**). The value of t_1/2ke_ (30.67 h) in this study was similar to that of a published report (23.14 h) both after orally administration (Ding et al., 2010), but higher than those in broilers (5.26 h), buzzards (4.11 h), beagle dogs (7.51 h), pigs (17.3 h), and turkeys (7.37 h) after intramuscular injection (Garciamontijano et al., 2001; Anadón et al., 2002; García-Montijano et al., 2003; Haritova et al., 2006). However, the value of C_max_ (0.55 μg/ml) after oral administration was lower than that after intramuscular injection, but similar to that (0.67 μg/ml) after oral administration reported by Haritova et al. (2006). The differences in these results were due to the routes of administration, species and dosing differences. Moreover, the value of AUC (14.67 μg.h/ml) was similar to that (11.37 μg.h/ml) reported in beagle dogs by Yohannes et al. (2015) and that reported in poultry (10.86 μg.h/ml) by Haritova et al. (2006).

Compared to plasma, the drug concentrations in the ileum content were significantly higher, with C_max_ and AUC of 11.28 μg/ml and 77.81 μg.h/ml, respectively, which were 20 and 5.3 times higher than those in plasma, respectively (**Table 1**). There were no other studies of the concentration of MBF in ileum content, and this was the first to evaluate the PK of MBF in the ileum content in this study. The difference in concentrations between plasma and intestinal contents could be due to the high lipophilicity of MBF; this could be the reason why the Cl_b_ (0.14 L/h) in plasma is much higher than that (0.026 kg/h) in intestinal contents in this study. These results revealed that MBF had a strong penetration ability for various tissues and easily accumulated in intestinal contents following oral administration. These characters were also similar to those of other studies of MBF in beagle dogs (0.17 L/h), pigs (0.21 L/h), and foals (0.34 L/h) (Vallé et al., 2012; Tohamy and El-Gendy, 2013; Yohannes et al., 2015). At the same time, MBF could also penetrate membranes and tissues, binding to solid parts of the ileum content. The drug concentration dissolved in the aqueous phase was the main activity and worth detecting. Due to the high drug concentrations in ileum content, the AUC (77.81 μg.h/ml) was much higher than that (22.56 μg.h/ml) in plasma by intravenous injection administration in the previously described report (Ding et al., 2010), the bioavailability was considered as 1 for calculation, and it also was reported in the published reports (Wang et al., 2016; Yan et al., 2017).

As a kind of concentration-dependent action for MBF, the parameters C_max_/MIC > 10 and AUC_0-24 h_/MIC > 125 were used as a threshold for the successful therapeutic outcome of fluoroquinolones against gram negative bacteria (Toutain et al., 2002). However, these thresholds may be different for different fluoroquinolones. There were differences in the immune status of target animals and pathogens. The published AUC_0-24 h_/MIC was 46 h for bactericidal action in PK/PD study of MBF against *Mannheimia haemolytica*, and 88 h for the bactericidal action of MBF against *Haemophilus parasuis* (Aliabadi and Lees, 2002; Sun et al., 2015). Therefore, it was of great importance to study the PK/PD indices of fluoroquinolones including MBF individually. In this study, the PD data were obtained from ileum content to predict dosage regimens since it was more clinically relevant than those from broth. The ratios of *ex vivo* C_max_/MIC, C_max_/MPC, AUC_0-24 h_/MIC, and AUC_0-24 h_/MPC were 5.64, 1.37, 23.08 h and 5.63 h, respectively in ileum content (**Table 2**). The inhibitory sigmoidal E_max_ model was used for PK/PD integration model and dosage prediction, and it showed a favorable correlation (0.995) between the observed and predicted antibacterial efficacy of MBF against *E. coli ex vivo* (**Figure 6**). The *ex vivo* AUC_0-24 h_/MIC ratios of MBF requiring bactericidal action and eradication of the clinical strain with MIC_90_ of 2 μg/ml were 23.54 and 27.18 h, which were similar to *in vivo* AUC_0-24 h_/MIC (23.08 h) achieved after oral administration (2 mg/kg). These results suggest that the recommended dosage of 2 mg/kg could guarantee clinical efficacy against infections associated with sensitive *E. coli* with an MIC_90_ of 2 μg/ml. Based on the Monte Carlo simulations, the predicted daily dose for 50 and 90% targets to achieve bactericidal effect were 1.22 and 1.33 mg/kg, respectively. The Monte Carlo simulation to predict dosage for clinical use had the advantage of taking into account the PK/PD parameters based on bacteriological outcome. Furthermore, it could set target percentage such as 90 and 50% for simulation models for all data in relation to incidence in pigs (Dorey et al., 2017). However, due to the animals’ immune system also being an important factor contributing to bacterial eradication, the bacterial endpoint *in vivo* conditions may differ from the predicted dosages *ex vivo* (Garrison et al., 2002; Yan et al., 2017).

## Conclusion

Most studies have demonstrated that the increasing antimicrobial resistance in gut flora was due to the misuse of antibiotics in unsuitable and irrespective administration routes (Nguyen et al., 2012; Zhang et al., 2013). The PK/PD model in the infection target site could provide more reasonable dosages. According to our PK/PD parameters *ex vivo*, the single doses required to reach bacteriostatic, bactericidal, and eradication activity for 90% target were 0.92, 1.33, and 1.53 mg/kg, respectively. These results demonstrated that the administered dosage (2 mg/kg) of marbofloxacin by oral administration daily could have an effective bactericidal effect against *E. coli*. Furthermore, it provided an alternative optimal dosage regimen (1.33 mg/kg for bactericidal and 1.53 mg/kg for eradication) and avoided the emergence of resistance for clinical veterinary use. However, the predicted dosage regimens should be validated in clinical practice to evaluate the treatment effect of infected pigs in future research.

## Author Contributions

JC conceived the study; JC and ZL designed the experiments; ZL, QL, and BY performed the experiments; ZL wrote the manuscript; QH, QZ, SY, KL, JX, and SZ improved the language. All authors reviewed the manuscript.

## Conflict of Interest Statement

The authors declare that the research was conducted in the absence of any commercial or financial relationships that could be construed as a potential conflict of interest.

## Abbreviations

AUC: area under the curve
AUC_0-24 h_: area under the curve from 0 to 24 h
bw: body weight
CFU: colony forming unit
Cl_b_: body clearance
C_o_: presented the initial concentration
C_max_: peak concentration
*E. coli*: *Escherichia coli*
E_max_: the Lg change in bacterial counts of blank sample
E_0_: the maximal antibacterial effect
EC_50_: the value to achieve 50% maximal antibacterial effect
Hill: the dose-response curve slop
Ke: elimination rate constant
MBC: minimum bactericidal concentration
MBF: marbofloxacin
MIC: minimum inhibitory concentration
MPC: mutant prevention concentration
MRT: mean residence time
N: the Hill coefficient
PK/PD: pharmacokinetic/pharmacodynamic
t_1/2ke_: elimination half-life
V_d_: apparent distribution volume
